# Supplementary material for: Role of Solvent Compatibility in the Phase Behavior of Binary Solutions of Weakly Associating Multivalent Polymers
Source: Biomacromolecules. 2021 Dec 4;23(1):349–64. doi: 10.1021/acs.biomac.1c01301 (PMC8753604; doi:10.1021/acs.biomac.1c01301)
Supplement: Supplementary file 1 — bm1c01301_si_001.pdf [file bm1c01301_si_001.pdf]

Role of Solvent Compatibility in the Phase Behavior of Binary Solutions of Weakly Associating Multivalent Polymers

Jasper J. Michels[1], Mateusz Brzezinski[1], Tom Scheidt[2], Edward A. Lemke[2], Sapun H. Parekh[1,3]*

[1]*Max Planck Institute for Polymer Research, Ackermannweg 10, 55128 Mainz, Germany*

[2]*Institute for Molecular Biology, Johannes Gutenberg University, Ackermannweg 4, 55128 Mainz, Germany*

[3]*Department of Biomedical Engineering, The University of Texas at Austin, 107 W Dean Keeton Street Stop C0800, Austin TX 78712, USA*

* *michels@mpip-mainz.mpg.de*

SUPPLEMENTARY INFORMATION

Contents:

Coupling the solvency of Polymer B and polymer-polymer interaction

Coupling the solvency of Polymer B and polymer-polymer interaction

Her we reproduce the calculations underlying Figure 6 (in the main document) but assume a change in solvency of Polymer B (χ_{BS}) to be coupled with a change in the non-specific interaction between the two polymers (χ_{AB}). A reasonable assumption seems that if the solvency of Polymer-B decreases, *e.g.* due to a stronger mutual attraction between B-monomers, its interaction with Polymer-A also becomes more repulsive. The calculations represented below maintained the assumption of full miscibility in the binary polymer melt ($\chi_{AB} < \chi_{AB,\mathrm{crit}}$ = 0.00583), though now with χ_{AB}varying between subcritical for $\chi_{BS}^{(0)}$ = 0.54 and zero for $\chi_{BS}^{(0)}$ = 0.40. Linear extrapolation to $\chi_{BS}^{(0)}$ = 0.25 then gives $\chi_{AB} < 0$. All values are listed in Table S1.

Table S1. *Input for the Polymer-B:solvent and Polymer-A:Polymer B interaction parameters at* T_{ref} *= 273 assuming a linear coupling. The second entry represents the same values as used for Figure 6 (main document).*

$\chi_{BS}^{(0)}$	$\chi_{AB}^{(0)}$
0.25	-0.0134
0.40	0
0.54	0.0125

The phase diagrams calculated (at T = 300 K) using the value in Table S1 are plotted in Figure S1. Upon comparison with Figure 6 in the main text, we encounter the largest differences in the segregative regime, though the overall shapes and trends remain unaltered compared to the case where $\chi_{AB}^{(0)}$ = 0 and constant. In general, in the associative regime the

differences are very small though still noticeable. We have indicated the changes compared to Figure 5 with the dark blue arrows. For $\chi_{BS}^{(0)} = 0.25$, the miscibility gap slightly widens in the associative regime (red frame), but contracts in the segregative regime (green frames). Interestingly, the opposite trend is observed for $\chi_{BS}^{(0)} = 0.54$, where a very significant widening is seen in the secondary segregative subregime (blue frame). In the intermediate cases (orange frames), the size of the miscibility gap remains unaltered but the overall tilt in the tie-lines becomes more positive/negative for better/worse Polymer B solvency.

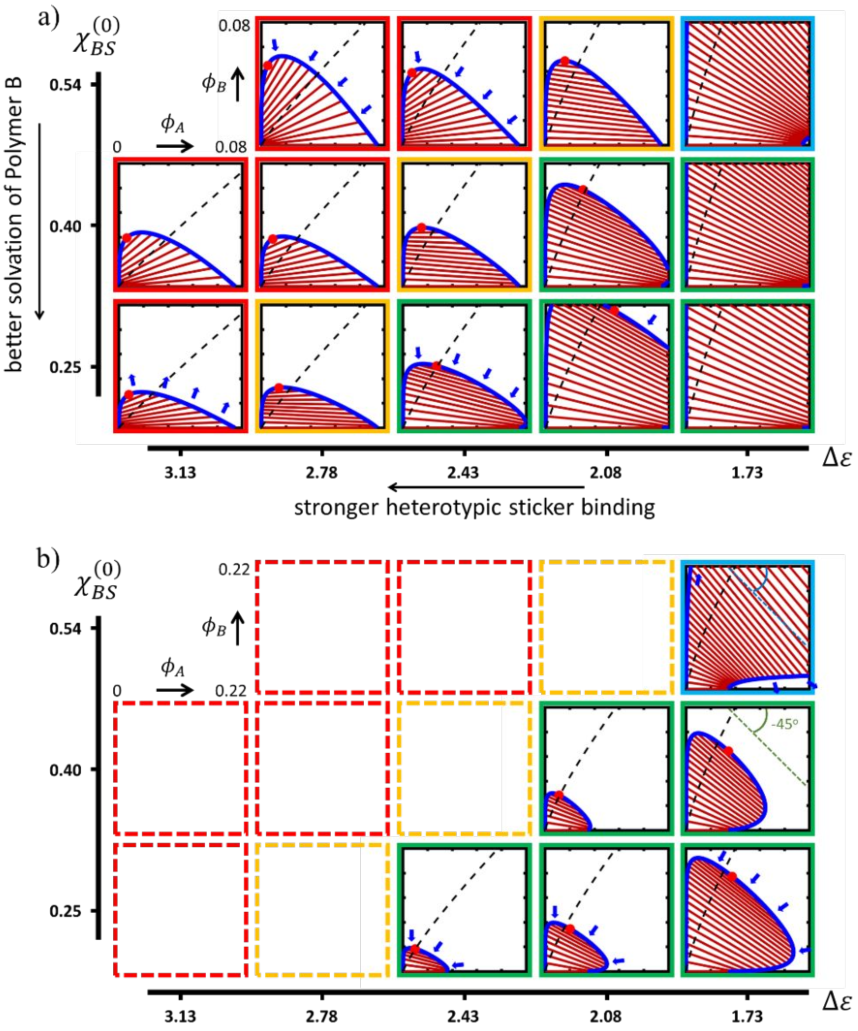

Figure S1. *Isothermal (T = 300 K) ternary phase diagrams (red symbol: critical point, blue line: binodal, brown lines: tie-lines), plotted as a function of the relative strength of the heterotypic (AB) sticker association and solvent quality for Polymer-B with $\chi_{AS}^{(0)} = 0.3$. The*

red, orange, and green/blue frames indicate the regimes of associative, neutral and segregative LLPS, respectively. The dashed black lines indicate the compositions for which $p_{AA} = p_{AB}^{(A)}$*. Panel b) shows the phase diagrams of the segregative regime on a more extended composition scale.*
